# Global health diplomacy: advancing foreign policy and global health interests

**DOI:** 10.9745/GHSP-D-12-00048

**Published:** 2013-03-21

**Authors:** Josh Michaud, Jennifer Kates

**Affiliations:** a Kaiser Family Foundation, Washington, DC

## Abstract

Attention to global health diplomacy has been rising but the future holds challenges, including a difficult budgetary environment. Going forward, both global health and foreign policy practitioners would benefit from working more closely together to achieve greater mutual understanding and to advance respective mutual goals.

Recently, there has been a remarkable surge of interest in the topic of “global health diplomacy” (GHD). Official GHD offices have been established at the World Health Organization (WHO) and at the U.S. Department of State, and offices within governments of many countries now have a broad set of new GHD responsibilities.^1-3^ Academics have begun to publish articles on the subject in greater numbers; more than 70% of all peer-reviewed journal articles on GHD since 1970 were published in the last decade, according to a recent analysis.^4^ While international engagement on health issues has a history that extends back to at least the 19^th^ century, the renewed emphasis is notable. What is driving this interest in—and support for—GHD, and what might it imply for the current and future practice of global health?

## WHAT IS GLOBAL HEALTH DIPLOMACY?

It is worth noting that, even with this growing level of interest, there is little agreement on how to define “global health diplomacy.”^5-6^ Generally, GHD refers to international diplomatic activities that (directly or indirectly) address issues of global health importance, and is concerned with how and why global health issues play out in a foreign policy context. GHD can encompass a broad set of activities and actors, such as formal country delegations holding bilateral and multilateral negotiations on health issues, a combination of governmental and nongovernmental actors negotiating on health-related issues, and, although often not considered “diplomacy” in the traditional sense, official or semi-official representatives of one country acting in a health capacity in another (see box for specific examples).

**Box. Types of Global Health Diplomacy Activities**

**Formal international bilateral and multilateral negotiations,** such as those that take place at the World Health Assembly and other multilateral forums and traditional negotiations between donor and recipient countries regarding official bilateral health assistance

- Negotiations around the WHO Framework Convention on Tobacco Control
- The U.S. President's Emergency Plan for AIDS Relief (PEPFAR) Partnership Framework agreements on HIV/AIDS between the U.S. government and partner countries

**Multi-stakeholder diplomacy** that often includes countries as well as non-state actors negotiating on health-related issues

- The Global Fund to Fight AIDS, Tuberculosis and Malaria and GAVI (formerly the Global Alliance for Vaccines and Immunization)
- The 2012 London Summit on Family Planning

**Interactions between health actors from one country acting in another country,** which include the activities of official and semi-official representatives from donor countries acting in recipient countries, for example the U.S. Agency for International Development (USAID), PEPFAR, or contracted NGO staff interacting with officials of the host country

- USAID country staff advocating inclusion of family planning services in Ghana's national health insurance program
- U.S. Ambassador calling for greater funding of child survival programs in Malawi's national budget

Source: Adapted from reference 4.

## ADVANCING BOTH HEALTH AND FOREIGN POLICY AIMS

Perspectives differ as to whether GHD is driven primarily by global health or foreign policy aims. Global health proponents, for their part, have mostly characterized GHD as a unique opportunity to raise the policy profile of global health. They perceive tying health issues to foreign policy and diplomacy as a recipe for more attention and resources. For example, the current WHO Director-General has characterized the new focus on diplomacy as signaling a “new era” for global health,^7^ while the Editor of the *Lancet* expects that such a diplomatic focus can move “foreign policy away from a debate about interests to one of global altruism.”^8^ On the other hand, many politicians and foreign policy practitioners emphasize how support for health programs can help achieve foreign policy goals through the application of “soft power” and the practice of “enlightened self-interest.” At times these two different viewpoints may align, but there are many examples when they do not, with foreign policy and national security goals often trumping global health objectives. Some examples include:

- Tightening intellectual property rights on drugs and vaccines instead of promoting ease of access to medicines^9-11^
- Promoting the production, sale, and trade of products detrimental to health (such as tobacco, alcohol, and junk foods)
- Using fake vaccination programs in the context of counter-terrorist operations that can jeopardize community trust in basic public health programs^12^

## WHY NOW?

A number of factors have contributed to the growing attention to the intersection between global health, diplomacy, and foreign policy. Several high-profile global health challenges, including HIV/AIDS and emerging infectious diseases such as SARS (severe acute respiratory syndrome) and pandemic influenza, came to be seen as direct threats to core national security and foreign policy interests, forcing senior policy makers to consider health issues in a new light, which had previously been relegated to a lower policy priority. Partly in response to this, but also in recognition of the need to address ongoing global health disparities and realize the Millennium Development Goals, donors have dedicated more resources to overseas health programs and created new channels of assistance, leading to greater policy attention and scrutiny.^13-14^

Further, global health has grown more interdisciplinary, with links increasingly being made between health and other areas such as international trade and intellectual property rights, agriculture, education, and the environment. In the process, health has moved onto diplomatic and foreign policy agendas.^15^ Another factor has been an unprecedented level of personal engagement and commitment shown by many political leaders and high-profile personalities, such as U.S. President George W. Bush, former U.S. Secretary of State Hillary Clinton, UK Prime Minister David Cameron, singer Bono, and Bill and Melinda Gates.^16-18^

High-profile global health challenges such as HIV/AIDS have been perceived as direct threats to national security and foreign policy interests.

## RECENT EXAMPLES

Even though much of the focus in GHD may be on carefully planned engagements between actors with explicit, joint interests and objectives, some GHD activities are undertaken in response to crises or to resolve unexpected issues that arise. These may draw in a range of different actors and encompass a variety of diplomatic activities depending on the situation at hand. For example, when politicians and community groups in several states in Northern Nigeria ceased supporting polio vaccination in 2003, international diplomacy efforts were undertaken to restart the polio campaign, bringing together the U.S. (with representatives from the State Department, the Centers for Disease Control and Prevention, and other agencies), the United Nations, WHO, the Organization of the Islamic Conference (currently called the Organisation of Islamic Cooperation), the African Union, Bill Gates, vaccine manufacturers, and others.^19^

In another example, a broad set of diplomatic actors engaged in a series of formal and informal negotiations with representatives of the Indonesian government when it refused to share H5N1 (avian influenza) virus samples with the Global Influenza Surveillance Network beginning in 2006. The Indonesian government was concerned that the country did not receive benefits from sharing these samples, but its actions threatened efforts to track the potential emergence of an H5N1 pandemic. The parties eventually resolved the immediate issue at hand, but discussions around benefit-sharing and pandemic influenza surveillance and prevention continue even today.^19^

Lastly, there has been growing recognition of the important role of U.S. ambassadors in health diplomacy, largely driven by the creation of PEFPAR in 2003, which brought significant new resources, not to mention a complex new program, to U.S. overseas missions.^21^ Indeed, the work of the new Office of Global Health Diplomacy at the State Department is focused squarely on the role of ambassadors, which “will be elevated as they pursue diplomatic strategies and partnerships within countries to foster better health outcomes.”^3^

## NEW WAYS OF WORKING

There are hallmarks to the practice of GHD that are worth noting. As the above examples indicate, non-state actors (including private companies, foundations and charities, NGOs, and civil society groups) can play an important role in GHD, a development which distinguishes this kind of diplomacy from the more traditional, formal negotiations between governmental representatives that characterized diplomacy historically. An additional dimension is the shift from a traditional binary or multipolar set of interactions among discrete government actors in formal settings towards a more dynamic “networked” approach to engagement, aided by instant communication capabilities and newly developed social media platforms and tools. Those engaging in GHD may be served well by taking these characteristics into consideration.^22^

Non-state actors can play an important role in global health diplomacy.

## NEW CHALLENGES

As of today, donor assistance for health has plateaued (and has shrunk in some cases)^23^ in the wake of the global economic crisis, causing countries to reassess their overseas assistance programs and seek ways to collaborate with and leverage other donors, while also seeking greater domestic commitments to health programs from low- and middle-income countries themselves.^24-25^ The continuing march of globalization and development has led to rising incomes and greater capacities in many previously poor countries, contributing to their desire to be seen as equal partners and not simply aid recipients. Navigating these trends going forward will require significant engagement from all sides, with attention to negotiation around issues of “country ownership,” health systems, and fostering equitable and efficient trilateral and multilateral partnerships. Meanwhile, players must now address a set of newly recognized health issues that face developing countries, such as non-communicable diseases and mental health, alongside the unfinished business of reducing illness and death from preventable infectious diseases and promoting maternal and child health.

Global health diplomacy requires attention to issues of country ownership and fostering equitable partnerships.

## A WAY FORWARD

Will GHD become a defining characteristic of the global health response in the next decade? Given recent trends, we might expect that it will continue to grow in importance. But in order to ensure international engagements on health and foreign policy are truly a win-win, both foreign policy practitioners and global health proponents need to engage more substantially. In an increasingly interdependent world, it is in the long-term interests of every country to have safe, prosperous, and healthy populations in partner countries, and the diplomatic community would do well to recognize that the global health agenda is a strong tool to achieve these goals. Likewise, global health experts could make greater efforts to understand and engage with foreign policy practitioners by keeping diplomatic leadership and embassy staff well informed of their activities and by clearly drawing the link between these activities and the broader policy objectives of foreign policy. Global health proponents also have additional opportunities to seek more visibility and to push for a seat at the table within the context of a broader set of international diplomatic issues with health implications, such as climate change and human trafficking.

Still, in light of the difficult budgetary environment faced by many donor countries and some of the inherent tensions between foreign policy and global health objectives, the extent and impact of GHD going forward remain difficult to predict. But if global health and foreign policy practitioners work together for greater mutual understanding and coordination, we can help to create conditions whereby all parties can benefit from the growing profile of GHD.
